# Male and female songs propagation in a duetting tropical bird species in its preferred and secondary habitat

**DOI:** 10.1371/journal.pone.0275434

**Published:** 2022-10-03

**Authors:** Amie Wheeldon, Katarzyna Kwiatkowska, Paweł Szymański, Tomasz S. Osiejuk

**Affiliations:** Department of Behavioural Ecology, Institute of Environmental Sciences, Faculty of Biology, Adam Mickiewicz University, Poznań, Poland; Claremont Colleges, UNITED STATES

## Abstract

Acoustic signals produced by animals must transmit through the environment to reach potential receivers and change their behaviour. Both the environment (vegetation, air properties, other animals) and the form of the signal affect the propagation process. Here we investigated how the transmission of different song types of a duetting songbird species inhabiting an extreme environment within African montane forest, varies between males and females as well as different types of micro-habitats. We hypothesised that male and female songs would have different transmission properties, reflecting known differences in signal form and function. We analysed signal-to-noise ratio (SNR), excess attenuation (EA) and tail-to-signal ratio (TSR) of songs of male and female Yellow-breasted Boubous (*Laniarius atroflavus*) that were played and re-recorded in a range of sites representing the species-typical habitats. We found significant effects of distance, site (habitat) and sex reflected in all three measures of sound degradation. The clearest, primarily distance-dependent pattern was found for SNR of songs propagated in level forest site. EA was substantially higher in shrubs than in forest habitats, while TSR reflecting longer echoes appeared at longer distances in forest sites. Thus, Yellow-breasted Boubou songs are better propagated in forests than in disturbed sites covered with shrubs. We found that all male song types used for broadcast singing propagated farther than female songs, with significantly higher SNR at all distances. The different male song types which are known to have different functions, also demonstrated a differentiated pattern of propagation reflecting their functionality. All signals that were tested propagated the furthest in the ideal condition described as forest with a level terrain. Signals degraded much faster during transmission through shrubs regrowing after forest burning. On this site, the differences in the propagation of male and female songs, as well as the differences between male song types, were relatively least pronounced. Transmission in typical mountain forest among streams and with substantial terrain variation revealed that degradation pattern in such habitat could be perturbed in a non-linear way. Streams acting as a source of high noise level also negatively affected transmission and may strongly limit the perception of birds staying close to them. However, stream noise did not affect sex differences in song propagation as was found for the site located in shrubs. Male songs showed more efficient transmission through all habitats (least in the shrubs) than female song. These differences were the result of male songs having a whistle structure that is better adapted for long-range propagation than the atonal, wideband frequency female vocalisations. Results support the idea that signals of males of the Yellow-breasted Boubous evolved under the pressure of long-range communication both with rivals and females, while females of the species are much more focused on within-pair communication or signalling together with their partner. The consequence of deforestation resulting in pushing back territories to the forest remnants along streams may be a shortening of the song’s active range, in particular, in females.

## Introduction

Animal signals evolved because they affected the behaviour of receivers [1]. In the case of acoustic signals, the distance between signallers and receivers may strongly affect a signal in regard to its attenuation and degradation. This then has a consequence for the characteristic of the signal at the place where it is received, and subsequently decoded by a receiver. Hence, factors affecting sound transmission through the environment are crucial for signal evolution and current characteristics [2]. For acoustic signals produced by animals and birds especially, the general rules of how the environment affects sound properties during transmission are relatively well known [3, 4]. On the other hand, the diversity of species and the specific signals they have evolved as well as the diversity of habitats (including human-induced habitats) make the propagation of signals a fruitful area of research, bringing important new knowledge and sometimes even unexpected conclusions. For example, a recent broad comparison of nearly 5000 passerine species revealed that after adding phylogenetic information to the model explaining song complexity, the peak song frequency is strongly negatively related to body size and that the cause of deviations from this allometric relationship is sexual selection and not, as one would expect, density of habitat [5]. Although this comparison challenges the acoustic adaptation hypothesis (AAH) [6], it must be viewed very sceptically. The disadvantage of this comparison may be the very coarse assignment of habitat density for each species, based on tree cover and a three-point scale of habitat openness. Without discounting the merits of Mikula’s impressive comparison [5], it is likely that birds’ adaptation for signal transmission are much more tuned to microhabitat scale, where specific places for singing as well location where receivers occur are of crucial importance [7, 8].

Most research addressing issues of sound propagation focus on testing the predictions of the acoustic adaptation hypothesis [6]. They usually concern adaptations of a single species (e.g., [9, 10]), comparisons of groups of species inhabiting similar habitats [11, 12] or compare different habitats [13, 14]. Some studies do address issues of sound propagation itself but also try to explore the problem of how propagation affects information encoded in the signal [10, 15, 16]. Despite the fact that there is probably no bird species that only produces a single type of acoustic signal, researchers rarely address issues related to within species signal variability in relation to their propagation characteristics and functions they fulfil. However, such studies shows that even different parts of the song in a species might be adapted to differentiated long- and short-range communication [17]. Birds usually have a set of structurally variable songs and calls which can be produced with both within- and between-species variation, from one to few thousands of different units like syllables or song phrases (types) [18]. Song evolved primarily as a long-range signal, which is produced as solo by male or females, as well as by pairs in duets [18, 19]. The majority of research on birds has been conducted in temperate regions of the Northern hemisphere where, typically, only males sing. Our knowledge is also biased toward males and specific climatic and habitat conditions [18]. Fortunately, progress in diversifying research has been done in recent years, and increasing (e.g. [20]). One of the issues relating to signal propagation that has the least focus is if male and female song in duetting species do propagate in a similar way or not, and how the functionally different signals of a species are degraded during transmission? Among the few studies on this topic, the recently published paper on Canyon Wren (*Catherpes mexicanus*) revealed that males and females of the same species have evolved different acoustic signals despite living in the same physical environment [21]. Therefore, sexual and social selection pressures have been attributed to being main factors responsible for sex-specific signal evolution trajectories in this species.

**Fig 1:**
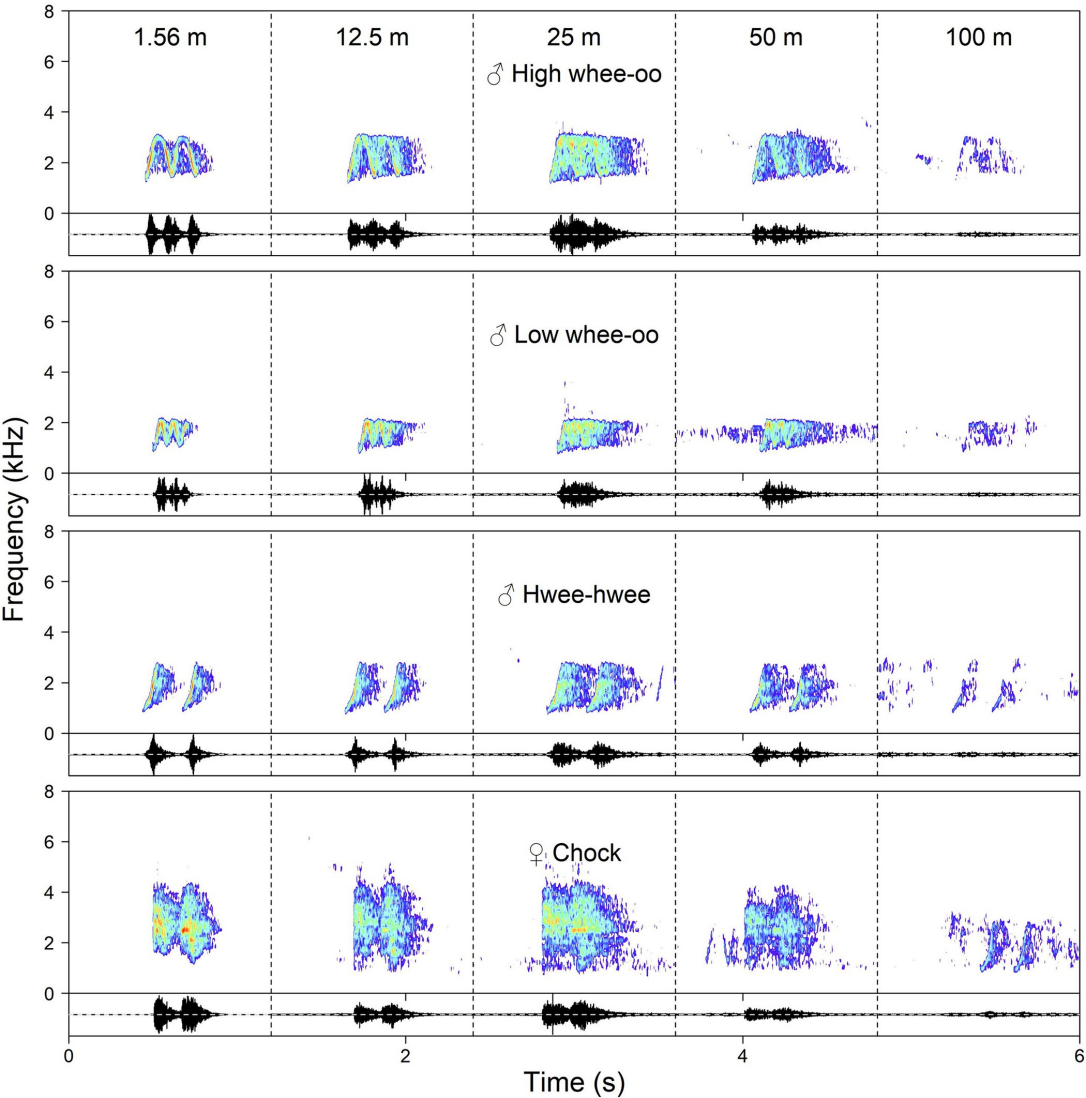
Male and female songs recorded at control distance (1.56 m) and after propagation on distances 12.5, 25, 50 and 100 m. Illustration prepared for the experiment conducted on a level terrain forest where the propagation sounds distortions were at the lowest level.

In this study we focused on a duetting species, which, in biological terms, suggests that propagation of its signals could be under strong and sexually differentiated selection pressure [22–24]. The study species is the Yellow-breasted Boubou (*Laniarius atroflavus*), an insectivorous bush-shrike, endemic to mountain rainy forest of E Nigeria and W Cameroon. It inhabits visually occluded, usually very dense (but locally patchy, including vertical dimension) habitats characterised by very variable environmental conditions in terms of temperatures, humidity (including direct rain) and wind [24]. Some of the accompanying animals like cicadas are also very noisy making vocal communication exceptionally difficult. Both sexes of the Yellow-breasted Boubou sing solos as well as in duets and their long-range vocalisations have strikingly different acoustic structures [22, 25] (Fig 1). They also produce a set of more or less sex-specific calls given in particular contexts, such as like for example alarming or excitement in short distance communication [22].

In this study we present the results of the Yellow-breasted Boubou songs propagation experiments conducted in their natural environment. As the species inhabit a quite various range of mountain forest micro-habitats, including those affected by human activity, we aimed at finding potential differences in sound propagation related to such changes. We were especially interested in quantifying differences in the propagation of male and female long-range signals between different habitat types. We have focused both on general differences of song transmission between sexes, but also on differences between male song types which are fully shared by all males withing the population and seem to be functionally differentiated [22, 23, 26]. According to the environment, model site was a typical mountain rainy forest growing on a level terrain at an altitude of around 2100 m asl. (S1 Fig). We compared propagation in this forest patch with comparably level shrubs area (forest regenerating after burning; S2 Fig). The third transmission experiment was done in hilly forest terrain between two streams and therefore increasing the general ambient noise level (S3 Fig). We also compared what is happening to the propagated sounds when they spread towards the direction to of natural noise source (stream) or from this source of noise. This experiment allowed for better understanding of communication constraints which appear when birds are forced to live in the remaining forest remnants that mostly include these streams. Different environments undoubtedly affect the propagation of sound signals. The easiest way to notice this is in environments that change abruptly. We have interesting examples where the changes resulted directly from anthropogenic noise, such as airport noise [27] or wind farms [28]. Yet, humans also affect or destroy the natural structure of the vegetation, which directly affects the parameters of sound transmission and the occurrence of other vocally active animal species, which after all, also changes ambient noise [29, 30]. Regardless of whether environmental changes are natural or human-induced, they may affect song behaviour and evolutionary diversification [31]. Finally, in this study, we focused more on the potential biological significance of sex specific differentiation of signal propagation as well as habitat and relief, rather than on detailed consideration of physical aspects of sound degradation per se.

## Methods

### Study area

The propagation experiment was conducted in the Bamenda Highlands of the Northwest Region of Cameroon. The study area was spread between 6°5’-6°8’N and 10°17’-10°20’E (altitude range 1900–2200 m asl The Bamenda Highlands are important hotspots of bird diversity and endemism in Africa [32]. As in many others forested areas in Africa the intensive logging has reduced formerly continuous forests to isolated patches in recent decades [33]. Nowadays, the vegetation covering the study area consists of mosaics of montane forest, forest remnants along streams, shrubby patches growing from formerly burnt forest, as well as grasslands and vegetable plantations below 1800 m asl. The study area is crossed by a network of streams which are a source of the natural noise generated by water falling on the rocks. Many bird species, including the Yellow-breasted Boubou, seem to prefer inhabiting areas with this increased noise live preferably in such environment being exposed to such ambient noise.

### Study species and its vocalisations

The Yellow-breasted Boubou is a monogamous, monomorphic, insectivorous bush-shrike that exhibit a sedentary lifestyle holding year round territories [34]. In the study area boubous start to breed at the end of the rainy season (October/November) and conduct following breeding attempts in the dry season. Males are much more vocally active than females and theirs solo vocalisations contribute the most to the overall species vocal activity [22, 24]. In the studied populations males have a fully shared repertoires of three whistle like song type phrases called High whee-oo, Low whee-oo and Hwee-hwee. Females produce different in structure vocalisations which different un structure to those of males and are described as being atonal and noisy. When females sing as a solo or in a duet, they produce phrases called Chock, Kee-roo or Chock series. Chock series are used for calling out their own mate and may initiate duet if the male is responding or remain as an unanswered female solo. Chock and Kee-roo are very similar in structure and the main difference is that Kee-roo has slightly prolonged and quiet ending. Both phrases are mostly used for responding to their male in a duet but are produced as a solo (more details in [22]. Birds sing loudly, typically between 86–92 dBA SPL at 1 m, but males can even produce songs reaching 103 dBA SPL at 1 m, while females are usually slightly quieter (4–6 dB) than males. These values were measured (CHY 650 Sound Level Meter, Ningbo, China) directly in the field for birds vocalising 5–15 m from the observer (distance measured with Leica DISTO 510 laser distance meter, Leica Geosystems, Heerbrugg, Switzerland). Males singing a solo or in a duet with a female produce phrases separated by a very regular interval of 1–2 s. Females usually follow each male phrase and overlap when performing a duet. Solo singing females produce s single phrase or sequences of phrases lasting a few-seconds and are characterised by a less constant repetition rate [22].

Yellow-breasted Boubous are sing both solos and in duets. We focused on the differences in propagation of solos in order to quantify the degradation of male and female specific signals. As we know, there is no structural differences in a solo or in a duet [26].

### Habitat and locations of experiments

The propagation experiments up to a distance of 100 m were conducted at three locations, which are later referred to as sites: FOREST, SHRUBS and STREAM (S1–S3 Figs). FOREST and SHRUBS sites were characterised by relatively level areas allowing for both the loudspeaker and microphones to be positioned at the same level with recorders (level above the ground and above sea level. Hence, for these two sites there was no other natural obstacles except vegetation due to the terrain. The STREAM site was placed within a hilly forest patch between two streams generating quite substantial noise. The extreme points of this transect (loudspeaker with playback and recording places) were located more than 10 m from streams, hence they were not under direct strong pressure of elevated noise. However, on an average the ambient noise level for this transect was higher than in the earlier two (Table 1). The transect was localised across the hill between two streams, with typical terrain variation, including ground bulges obstructing the path of the propagated sound. The transmission experiment on the STREAM site reflected the most typical natural environment of the study species, which inhabit forest typically located on slopes.

**Table 1:** Characteristics of propagation experiments with the Yellow-breasted Boubou (*Laniarius atroflavus*).

Characteristics of propagation experiments	Names of propagation experiments used in the manuscript				
	FOREST	SHRUBS	STREAM	STREAM FROM	STREAM TO
Site (habitat and terrain)	level terrain forest	level terrain shrubs, early stage of forest regeneration	hilly terrain forest located between streams	hilly terrain forest, close to stream	hilly terrain forest, close to stream
Total transect distance	100 m	100 m	100 m	25 m	25 m
Coordinates of speaker position	6.0904 N, 10.2960 E	6,0901 N, 10.2960 E	6.0901 N, 10.2954 E	6.0906 N, 10.2950 E	6.0905 N, 10.2950 E
Height asl (m) of propagation point ± vertical levelling of transect (m)	2141 ± 4 m	2059 ± 6 m	2026 ± 17 m	2042 ± 7 m	2035 ± 7 m
Date of experiment	29 Nov 2017	30 Nov 2017	1 Dec 2017	1 Dec 2017	1 Dec 2017
Time of experiment	6:51–9:25	6:50–8:47	6:53–9:21	6:53–9:21	6:53–9:21
Temperature (°C)	10.8–14.7	8.0–19.8	8.3–12.6	8.3–12.6	8.3–12.6
Humidity (%)	52–80	56–72	60–73	57–66	57–66
Ambient noise (dBA)	34–39	31–35	39–40	56–58	55–58

The last propagation experiment addressed the question of how direct stream noise affects the communication system depending on location of the signaller and receiver. Noise of a stream is common in Yellow-breasted Boubou habitat but is patchy in distribution both in space and in time. The STREAM transmission described above simulated degradation of the song in a forest patch with increased ambient noise due to neighbouring streams. However, the STREAM transect was located at some distance from the streams’ banks, hence both loudspeaker and microphones were not within the area of the loudest noise caused by falling water. In the last experiment we conducted propagation in the same area as the STREAM experiment but in different ‘micro’ locations. We imitated two situations when (1) a singing bird is located close to the stream (and source of noise), with the receiver being 25 m from the signaller, and (2) a singing bird is located ca. 25 m perpendicular to the stream while the receiver is located right next to the stream. These experiments are further referred as STREAM FROM and STREAM TO. The first point was located (horizontally) ca. 2 m from a loud stream (58 dB) and the second point was located 25 m to it (55 dB), perpendicular to the stream bank. Between these two points there were no terrain obstacles so only the stream noise and vegetation were the factors. In this case, the point closer to the stream was in a noisy environment and so such a location could be compared to a bird singing while moving along a stream. These last two experiments used the same two mentioned above points, and we only swapped the positions of the speaker and microphone for the STREAM FROM and STREAM TO tests. All three propagations located in the stream vicinity were conducted on the same day (Table 1).

Weather conditions and ambient background noise were measured at the beginning and end of each playback distance-session. In all three sites tested, the temperature and humidity increased during the time when transmissions were done (details in Table 1), but these differences were not statistically significant (ANOVA, temperature: *F*_2,21_ = 2.5, *P* = 0.1066; humidity: *F*_2,21_ = 0.13, *P* = 0.8794). The ambient noise varied between habitats: 34–39 dB(A) in FOREST, 31–35 dB(A) in SHRUBS, 39–40 dB(A) for STREAM, and 55–58 dB(A) for STREAM TO and STREAM FROM.

### Propagation experiment and test sounds

To assess the modifications of signals during propagation through the habitat, the songs of the Yellow-breasted Boubou were broadcasted, repeatedly and re-recorded several times from different distances in such a way that sounds propagating between the speaker and microphones crossed similar but not the same vegetation. Propagations were conducted during morning hours over the three following days, on 29 and 30 November and 1 December 2017, which could be considered as the peak of both breeding attempts and vocal activity [24].

Vocalisations used for propagation were recorded in the same area in 2014–17 with Marantz PMD660 and PMD661 (Marantz, Kanagawa, Japan) (frequency response: 20–22 000 Hz) portable recorders and Sennheiser ME 67 (Sennheiser electronic GmbH & Co. KG, Wedemark, Germany) microphones (frequency response: 40–20 000 Hz ± 2.5 dB) with windscreens, and all recordings selected were done within 3–6 m of the singing bird. Sounds were recorded as 48 kHz / 16-bit pulse code modulation (PCM) wav files.

We used high quality samples of Yellow-breasted Boubou songs recorded from 24 males (4–20 per male). We used 159 song phrases of High whee-oo type from 11 males (4–20 per male); 57 Low whee-oo song phrases from 6 males (5–14 per male); and 68 Hwee-hweee song phrases from 6 males (4–17 per male). For females, we used 36 of Chock song from 4 females (4–15 per female). In the case of female song, we limited our test to Chock song due to its similarity to the Kee-roo song and the lack of a sufficient number of good samples of the Kee-roos, the final part of which is very quiet [22]. Vocalisations from each individual were saved and later played back at their natural rates (i.e., in natural series) and there were pauses a few seconds long between songs of consecutive individuals to allow for the analysis of the background noise. The peak amplitude of each playback sample was prepared to match 90 ± 3 dB(A) (SPL at 1 m), which is within the natural levels the species.

### Experimental set-up and field recording

The microphones for rerecording of the tested signals were placed at 12.5, 25, 50 and 100 m from the speaker. Vocalisations recorded with microphones at these positions are referred to as propagated sounds. Recording at each distance was done sequentially but within a relatively short period (see Table 1 for details) and in random distance order. The different recording points were located on the same straight line to the speaker. The orientation of the microphone’s best sensitivity axis was always perpendicular to the speaker diaphragm and set up with the GPS find point function for averaged position of the speaker.

Additionally, all test sounds were recorded in an open and quiet area at ca 1.5 m from the speaker to prepare control sounds to be compared with the propagated sounds during the calculation of response measures (see the section ‘Sound analysis and response measures’ below for details). The speaker and the microphones were placed ca 2 m above ground level. Such a height reflected the natural song post location of the study species, with the exception that it may also vary greatly (own pers. obs.).

Signals were emitted by a MacBook Air (model: MacBookAir6,2, Apple Corp.) as PCM WAV files (48 000 Hz / 16 bits) connected to a UE Boom 2 (Ultimate Ears, Irvine, CA, USA) loudspeaker with a 9 W amplifier (frequency range 90–20 000 Hz and linear frequency response within species-specific frequency range). Recordings of propagated and control sounds were made with mentioned earlier Sennheiser ME 67 microphone and Marantz PMD661 recorder.

### Sound analysis and response measures

Signal degradation and attenuation during propagation through the natural environment were determined using the program SIGPRO v3.23 [35]. Transmission parameters were estimated by comparing control sound and propagated sounds according to an established protocol [7, 36, 37]. The tested sounds were not masked by transient noise of the same frequency. All recorded sounds were individually filtered in the signal frequency ranges characteristic for a given type of song deduced from the respective spectrogram. This individual approach allowed for keeping the structure of compared songs unchanged by this manipulation. The background noise (*E*_*noise*_) was measured immediately before each analysed sound in the quietest place only with stationary noise without echo of previous sound. To actually compare the control and propagated sounds, they were aligned in time by maximizing the cross-correlation function between them. The matching of model and observation signals allowed for the determination of the quantification of the signal energy (*E*_*y*_) and enabled us to calculate the signal-to-noise ratio (SNR), the excess attenuation (EA), the tail-to- signal ratio (TSR).

The signal to noise ratio was calculated as SNR = 10 log ((*E*_*y*_—*E*_*noise*_)/ *E*_*noise*_) [7] and allowed us get information about the net effect of masking and attenuation of acoustic signals. The excess attenuation was calculated as EA = – 20 log(*k*)–A [7]. EA is a parameter informing about the energy loss of the acoustic signal over the values provided by spherical spreading (6 dB per doubling the distance), i.e. Attenuation (A). Ratio *k* is the ratio of the energy of the model signal and the signal observed at a given distance. SNR and EA are usually negatively correlated, and both provide information about the potential transmission range of sounds. The tail to signal ratio was calculated as TSR = 10 log ((*E*_*tail*_ − *E*_*noise*_)/(*E*_*y*_− *E*_*noise*_)) [36]. The tail of echoes (*E*_*tail*_) is characterised by the gradual disappearance of the extension of the signal.

Measurements at the distance of 100 m were obtained only for male’s songs in the forest habitat. Female’s songs in forest habitat and all vocalisations in other analysed environments propagated at the distance of 100 m were degraded and attenuated at level preventing reliable comparison with the control sounds. Some sounds recorded were strongly masked by sounds of animals that were excluded from the analysis.

### Data analysis

We compared the song degradation measurements among the species’ vocalisations at different distances with the linear mixed models, which enabled the inclusion of repeated measurements of sound of the same individual as a random factor. This method allows for control for potential non-independence among vocalisations of the same individual. We ran several models explaining SNR, EA and TSR variation with sex, distance and site (habitat) and all two-way interactions as predictors. To test differences between male song types, we ran separate models limited to male songs propagation experiment. All statistics were calculated with lmer4 package [38] for the R environment (v.3.6.3, R Foundation for Statistical Computing). All P-values are two-tailed, and means are given with ±SE if not stated otherwise.

### Inclusivity in global research

Additional information regarding the ethical, cultural, and scientific considerations specific to inclusivity in global research is included in the S1 Checklist.

### Permits

The research presented here adhered to all local and international laws. Institutional approval was provided by the Local Ethical Committee for Scientific Experiments on Animals, University of Life Sciences, Poznań (permission no. 16/2015) and the Polish Laboratory Animal Science Association (certificate no. 1952/2015 to Tomasz Osiejuk) conforming to Directive 2010/63/EU.

## Results

### Differences in song propagation between sites reflecting habitats

**Fig 2:**
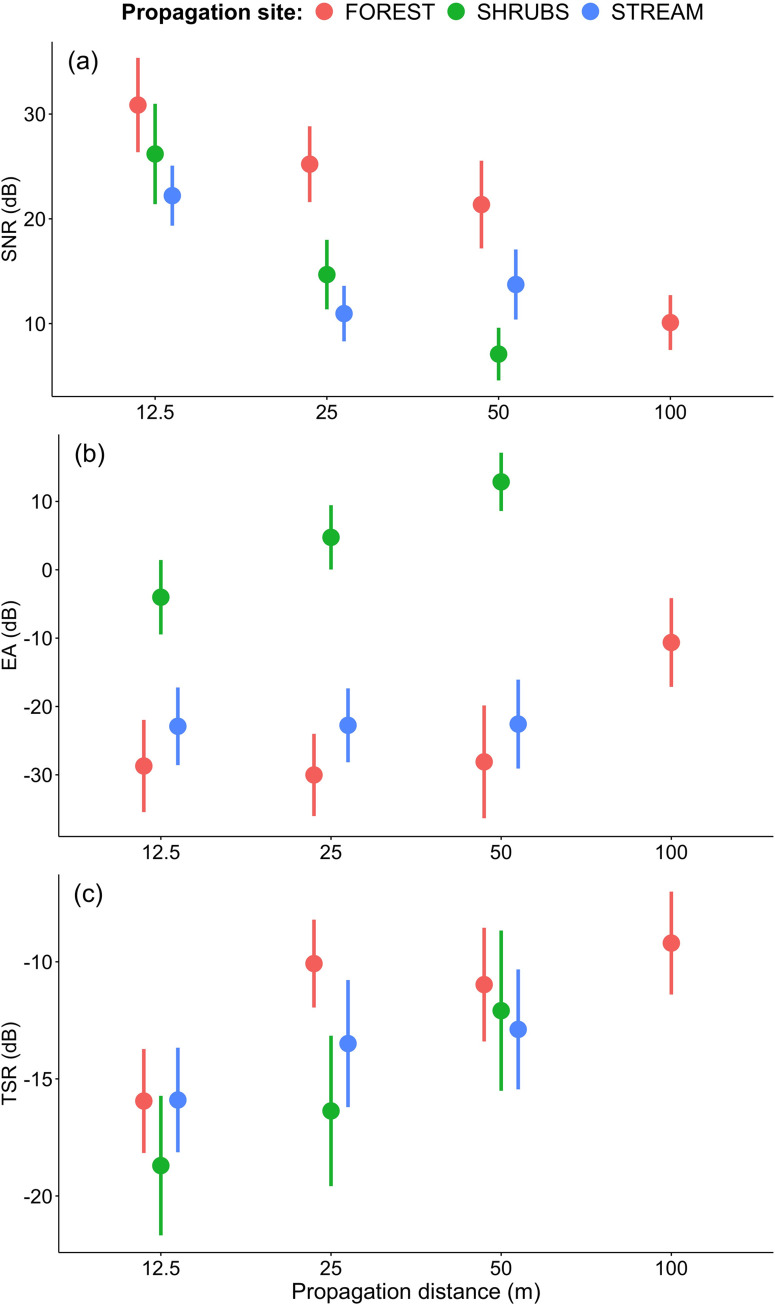
Signal-to-noise ratio (SNR±SD dB), excess attenuation (EA±SD dB) and tail-to-signal ratio (TSR±SD dB) for combined male and female song phrases in the forest, shrubs and along the stream.

We found that male and female songs degraded significantly differently with distance and site (Fig 2, Tables 2–4). Only in the case of FOREST transmission were we able to properly measure degradation of sound samples propagated to 100 m from the source. In the case of transmission in SHRUBS and STREAM it was not possible as the signals were too degraded after such a distance (Fig 2). Yellow-breasted Boubou songs propagated significantly worse in shrubs in comparison to level terrain forest at every distance that was compared (Fig 2). SNR was ~5 dB lower in SHRUBS than in FOREST as close at as 12.5 m from the loudspeaker and this discrepancy increased to ~14 dB at 50 m, where it was still possible to measure degradation of songs transmitted in shrubs. Characteristically, EA was substantially higher in SHRUBS than in both forest sites for each distance (Table 3, Fig 2). An irregular degradation pattern was found for the STREAM transmission, where SNR was on average larger after 50 m than for 25 m distance, and in both cases SNR values were more like the transmission in shrubs than in forest (Fig 2). Differences in TSR between sites were significant. The pattern was not so clear-cut as with SNR and EA, but differences in TSR reflected longer song echoes remaining at longer distances in both forest sites compared to SHRUBS (Table 4, Fig 2).

**Table 2:** Result of the general linear mixed model for signal-to-noise ratio (SNR) of propagated songs of the Yellow-breasted Boubou (*Laniarius atroflavus*). Main and two-factor interactions effects are presented for the following source of variation: sex (male, female), site (transect FOREST, SHRUBS, STREAM) and distance (12.5, 25, 50 and 100 m). Repeated measurements of songs of the same individuals are included as a random factor.

Source of variation	Coef.	Std. Err.	t	P
Main effects				
Sex	8.31	1.320	6.29	< 0.0001
Site	-5.70	0.357	-15.97	< 0.0001
Distance	-7.14	0.361	-19.78	< 0.0001
Two-factor interaction effects				
Sex × Site	-0.76	0.312	-2.45	0.0145
Sex × Distance	-0.43	0.323	-1.28	0.2008
Site × Distance	0.66	0.098	6.78	< 0.0001

**Table 3:** Result of the general linear mixed model for excess attenuation (EA) of propagated songs of the Yellow-breasted Boubou (*Laniarius atroflavus*). Main and two-factor interactions effects are presented for the following source of variation: sex (male, female), site (transect FOREST, SHRUBS, STREAM) and distance (12.5, 25, 50 and 100 m). Repeated measurements of songs of the same individuals are included as a random factor.

Source of variation	Coef.	Std. Err.	t	P
Main effects				
Sex	-14.16	3.458	-4.09	< 0.0001
Site	4.05	1.120	3.35	0.0008
Distance	3.41	1.230	2.77	0.0055
Two-factor interaction effects				
Sex × Site	1.63	1.052	1.55	0.1215
Sex × Distance	1.66	1.102	1.51	0.1321
Site × Distance	-1.22	0.334	-3.66	0.0002

**Table 4:** Result of the general linear mixed model for tail-to-signal ratio (TSR) of propagated songs of the Yellow-breasted Boubou (*Laniarius atroflavus*). Main and two-factor interactions effects are presented for the following source of variation: sex (male, female), site (transect FOREST, SHRUBS, STREAM) and distance (12.5, 25, 50 and 100 m). Repeated measurements of songs of the same individuals are included as a random factor.

Source of variation	Coef.	Std. Err.	t	P
Main effects				
Sex	-4.03	0.872	-4.63	< 0.0001
Site	-0.84	0.225	-3.75	0.0002
Distance	2.72	0.227	11.95	< 0.0001
Two-factor interaction effects				
Sex × Site	0.50	0.196	2.53	0.0113
Sex × Distance	-0.06	0.203	-0.31	0.7562
Site × Distance	-0.20	0.061	-3.32	0.0009

We found a significant effect of Sex × Site and Site × Distance interactions on SNR, indicating that female songs degraded faster than male songs with distance, as well as in habitats represented by SHRUBS and STREAM transmission, in comparison to level terrain FOREST. As could be expected, the analysis of EA changes with propagation distance reveals a negatively correlated pattern in comparison to SNR (Table 3). However, the difference in excess attenuation between male and female songs was clearly larger than for SNR. Energy loss measured by EA was much more substantial in shrubs than in both forest habitats, resulting in a significant effect of Site × Distance interaction. Hence, the energy loss of song was larger for females and for dense shrubs. We also found that the quality of sound assessed by the TSR was the most strongly affected by the distance, while significant differences between sexes and habitat differences still existed (Table 4). The significant effect of Site × Distance interaction on TSR again indicates that songs longer kept better quality in the forest than in shrubs.

### Degradation of different song types

#### Male and female songs in different sites

**Fig 3:**
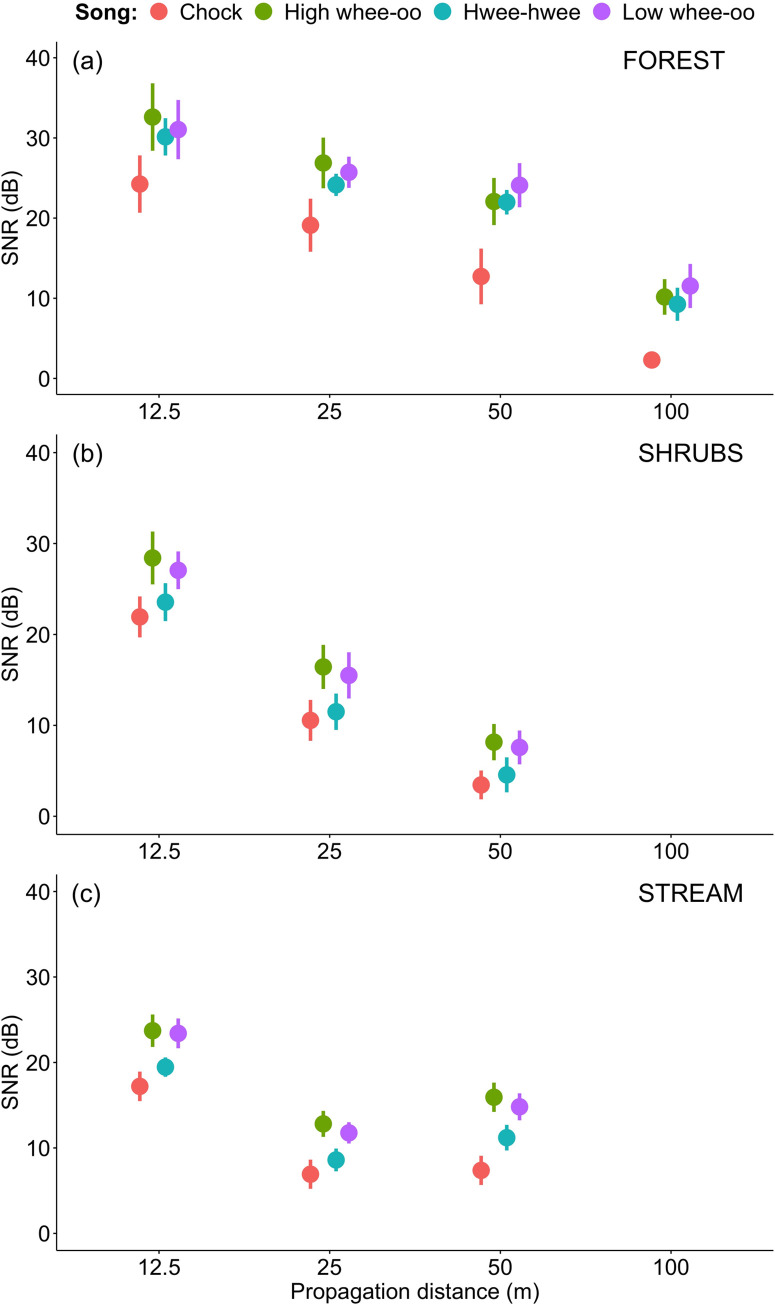
Comparison of signal-to-noise ratios (SNR±SD dB) for male (H–High whee-oo, L–Low whee-oo, W–Hwee-hwee) and female (C—Chock) song types propagated in three different sites: (1) the level terrain forest habitat type (FOREST), (2) the shrubs habitat type (SHRUBS), and (3) the hilly terrain forest located between streams habitat type (STREAM).

**Fig 4:**
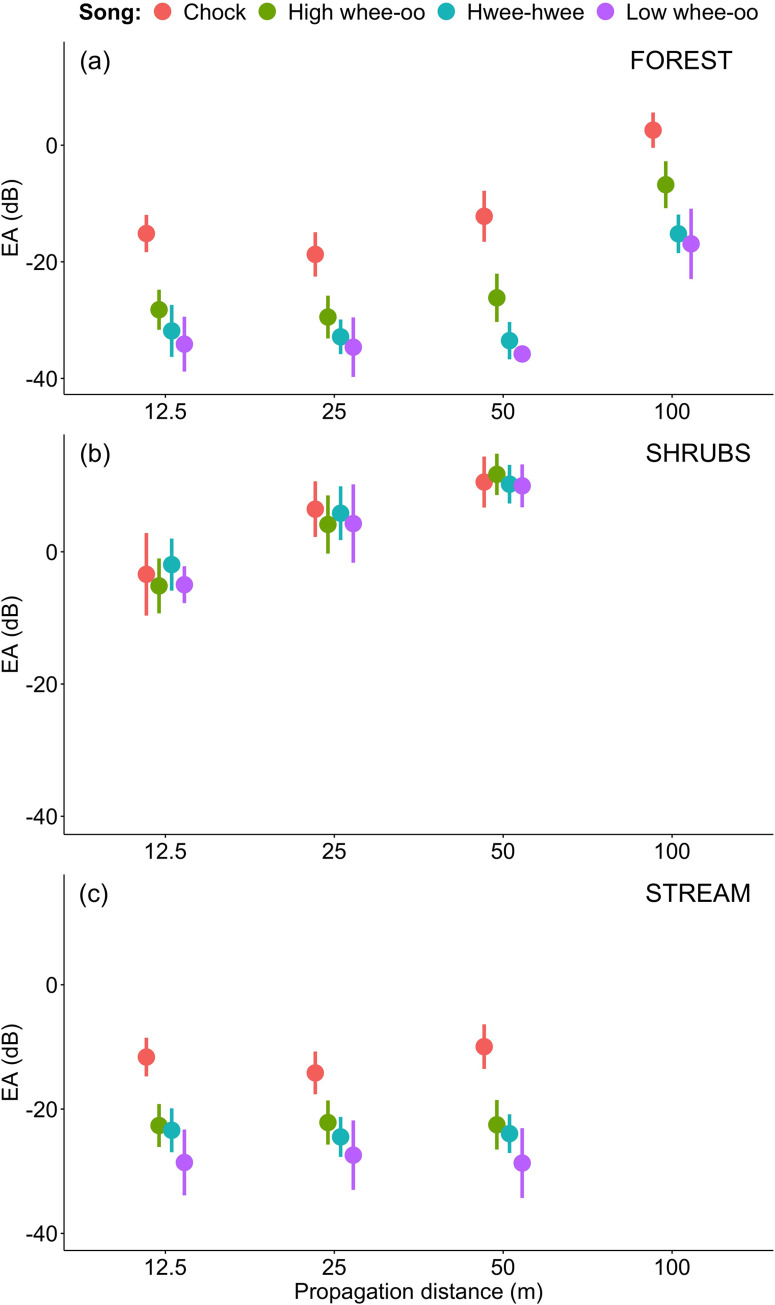
Comparison of excess attenuations (EA±SD dB)) for male (H–High whee-oo, L–Low whee-oo, W–Hwee-hwee) and female (C—Chock) song types propagated in three different sites: (1) the level terrain forest habitat type (FOREST), (2) the shrubs habitat type (SHRUBS), and (3) the hilly terrain forest located between streams habitat type (STREAM).

**Fig 5:**
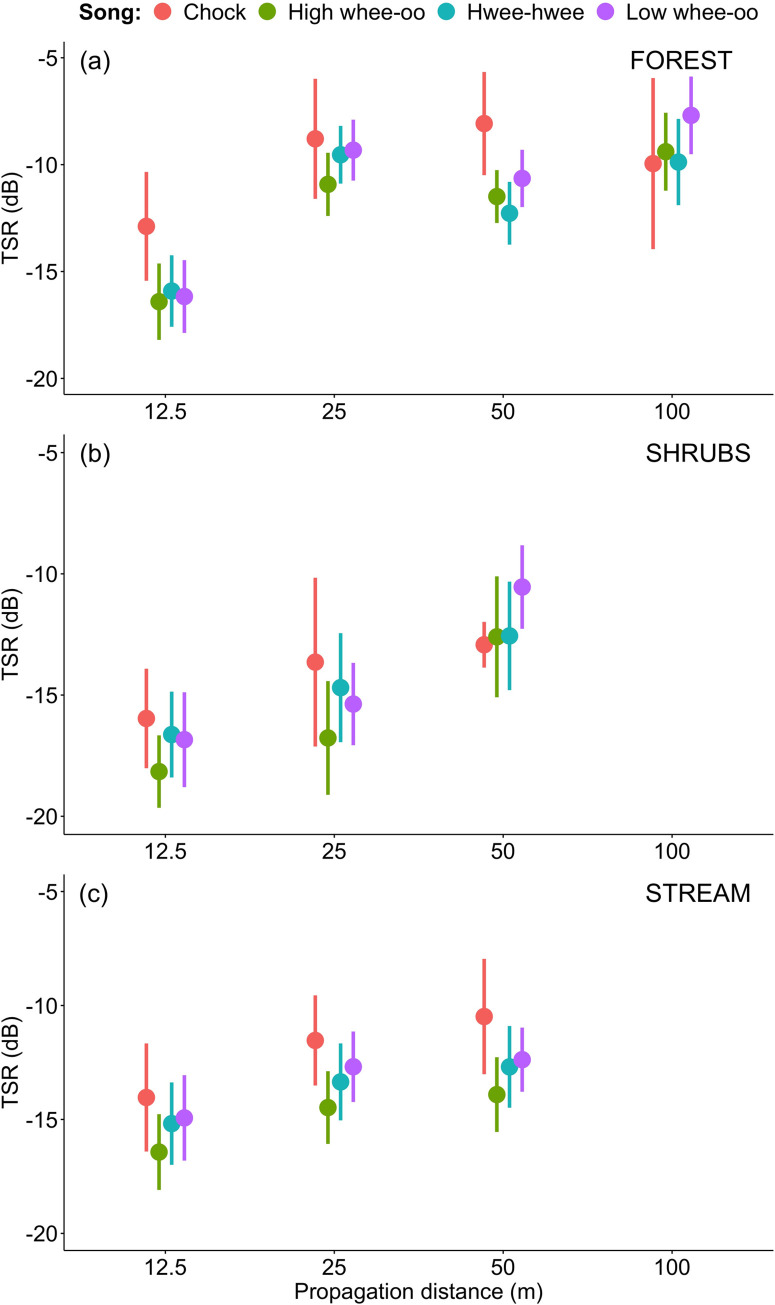
Comparison of tail-to-signal ratios (TSR±SD dB) for male (H–High whee-oo, L–Low whee-oo, W–Hwee-hwee) and female (C—Chock) song types propagated in three different sites: (1) the level terrain forest habitat type (FOREST), (2) the shrubs habitat type (SHRUBS), and (3) the hilly terrain forest located between streams habitat type (STREAM).

In the next step we compared male and female songs degradation in different sites. The sex differences seem to be rather consistent with all three male whistle song types transmitting always substantially better than female songs (Tables 2–4, Figs 3–5). The differences between male song types were relatively small at each distance if compared to female songs transmitted in FOREST (Figs 3–5). In SHRUBS male and female songs degraded during propagation in a more similar manner (see Figs 3–5). Although female songs still degraded the most, the male Hwee-hwee songs propagated only slightly better by meaning of differences in SNR (Fig 3). Again, a very interesting pattern was found for the STREAM transmission in a hilly forest patch among two streams. Male songs propagated better than female songs and female songs were very seriously distorted as quickly as 25 m from the speaker. In general, the decrease in SNR between 12.5 and 25 m was the most substantial for the STREAM transmission (Fig 3).

### Differences in degradation between male song types

All male song types propagated much better than female songs. In the forest habitat (FOREST), even after 100 m for all three male song types SNR was still around 10–12 dB, while for females it was only around 2 dB (Fig 3). The whistle structure of male song did not help too much in shrubs (SHRUBS), with male vocalisations (especially Hwee-hwee songs) degrading only slightly less than female Chocks (Fig 3). On the other hand, male song types also degraded significantly differently (details in S1 Table). In the forest with more noise from streams and terrain obstacles (STREAM) High wee-oo and Low wee-oo song types propagated better than Hwee-hwee song type, characterised by the widest band and double whistle structure (Fig 5). However, in terms of values, these differences were relatively small compared to the female Chocks (compare Figs 3–5).

### Stream noise

**Fig 6:**
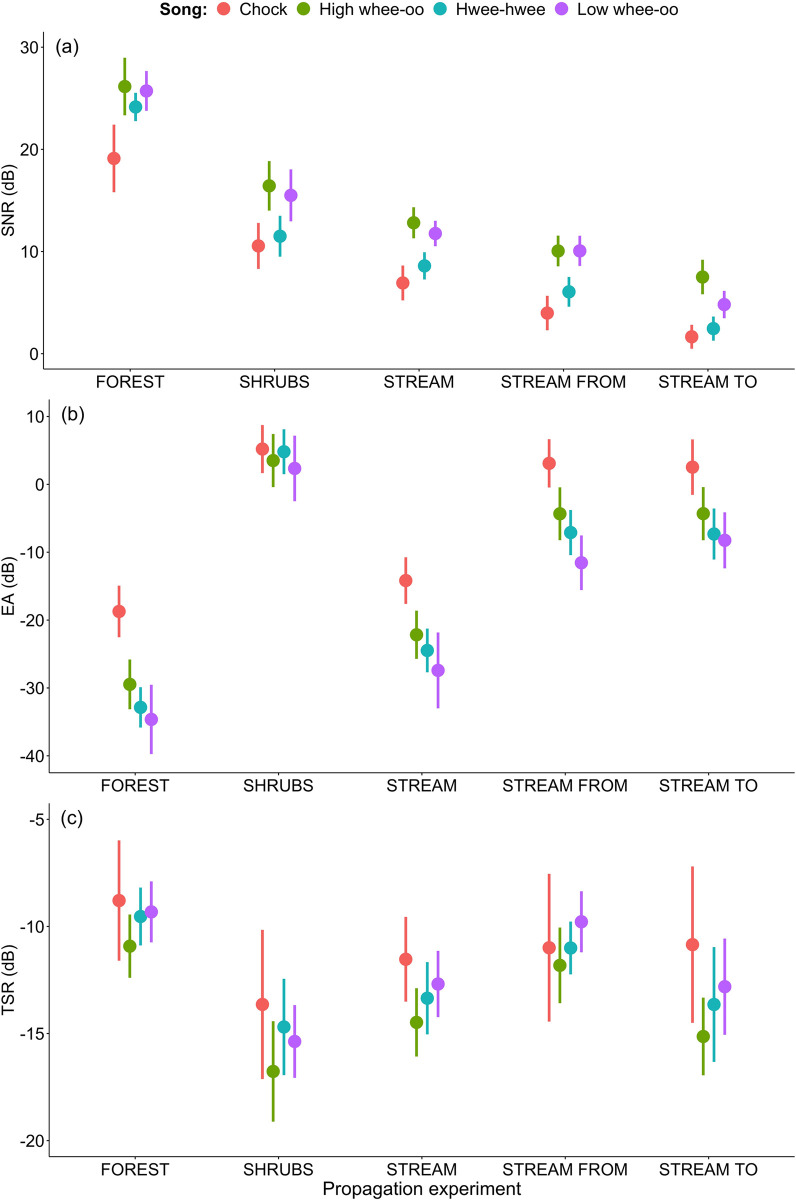
Signal-to-noise ratio (SNR±SD dB), excess attenuation (EA±SD dB) and tail-to-signal ratio (TSR±SD dB) measured after 25m of propagation for male (High whee-oo, Low whee-oo, Hwee-hwee) and for female (Chock) songs—comparison of all propagation experiments.

The last two propagations (STREAM FROM and STREAM TO) revealed that the location of receiver and sender of the signal in relation to a substantial source of noise is also important (Table 5, Fig 6). The distance between loudspeaker and microphone in these two propagation experiments was only 25 m and there were no serious obstacles between them. We found that if the receiver is located closer to the stream (i.e., source of substantial noise) the signal arriving to it is characterised by significantly lower SNR (~ 5 dB) in comparison to opposite situation (Table 5). Hence, the net effect of masking and attenuation of acoustic signals is strongly affected by close stream noise and the precise location of sender and receiver is crucial. On the other hand, the EA and TSR did not differ significantly between STREAM FROM and STREAM TO transmissions (Table 5, Fig 6B and 6C) suggesting a weaker effect of location on energy loss and quality of sound revealed by tail to noise ratio. Simultaneously, we found significant differences between male and female songs in all sound degradation measures (Table 5) suggesting a stronger effect on female than male vocalisations for a receiver located closer to a stream.

**Table 5:** Result of the general linear mixed models for signal-to-noise ratio (SNR), excess attenuation (EA), tail to signal ration (TSR) of propagated songs of the Yellow-breasted Boubou (*Laniarius atroflavus*). Main and two-factor interaction effects are presented for the following source of variation: sex (male, female), location (transmissions STREAM FROM and STREAM TO). Both transects were done on a distance of 25 m only, close to the stream noise and with opposite location of loudspeaker and microphone in each transmission.

Source of variation	Coef.	Std. Err.	t	P
*SNR (dB)*				
Main effects				
Sex	14.85	1.909	3.89	<0.0001
Location	-2.72	0.338	-8.05	<0.0001
Two-factor interaction effect				
Sex × Location	-0.65	0.356	-1.83	0.067
*EA (dB)*				
Main effects				
Sex	-16.27	4.462	-3.64	0.0003
Location	0.90	0.862	-1.04	0.297
Two-factor interaction effect				
Sex × Location	1.62	0.91	1.78	0.075
*TSR (dB)*				
Main effects				
Sex	11.85	2.835	4.18	<0.0001
Location	-0.49	0.584	-0.84	0.3993
Two-factor interaction effect				
Sex × Location	-3.09	0.614	-5.03	< 0.0001

We present on Fig 6 the degradation of male and female songs as measured by SNR, EA and TSR for 25 m propagation distances and all experiments conducted together. The sex differences in song degradation are obvious and consistent for all habitats. Surprisingly, the differences between habitats at a relatively small distance of 25 m may reach as much as ~20 dB of SNR. Despite observing significant differences between level terrain forest and shrubs, the shape of terrain, stream vicinity and location in relation to stream noise also seem to be very important when considering song degradation.

## Discussion

We tested differences in male and female Yellow-breasted Boubou song degradation during propagation in three sites representing their natural and human-altered habitats. We found significant and large differences between male and female songs, with songs of males propagating much better. The male song types also degraded differently, but these differences were not as substantial as between sexes. Song of the study species carried better in forest site. However, heterogenic mountain environment with its variable relief and noise of streams may affect transmission in a non-linear way making the right choice of place for singing (concerning the stream noise too) even more crucial than in homogenous habitat. Shrubs covering burnt forest affected signal propagation negatively and blurred differences between sexes typical for the forest sites.

### Song propagation in sites representing different habitats

#### Mountain rainy forest as a habitat for song transmission

The Yellow-breasted Boubou is a species endemic to mountain rainy forest inhabiting elevations from as low as 700 m asl on Mt Cameroon slopes, but in general preferring higher levels reaching 2,900 m asl [39]. Forest habitats growing at such different altitudes and at locally diversified locations, both in a macro-scale (various mountain ranges) and micro-scale (different relief etc.) represent very variable environment for sound transmission. The propagation experiment we conducted in level terrain forest (FOREST) should be considered the ideal condition (in the sense of a reference point), in terms of habitat for sound propagation, that the species may meet. A flat surface allows for forest stratification with a closed canopy between 20–25 m and a relatively open understory (like montane cloud forest with *Carapa* sp. trees [40]. Hence, songs propagated in FOREST had a relatively small amount of obstacles such as tree trunks and foliage as at the transmission level vegetation cover was below 20% at the height of the transmitted sound. Moreover, such a habitat is relatively free of spatial and atmospheric heterogeneities in comparison to open areas, which strongly affect signal degradation [41]. Within the study area, such fragments of forest were relatively rare, and we had a problem finding a flat 100 m transect for the FOREST transmission. Much more realistic in terms of availability of habitats and relief was the STREAM transmission experiment, with both diversified elevations of recording locations, stream noise and more patchy vegetation in the understory.

The difference in song degradation between FOREST and STREAM was large. Firstly, in level terrain forest songs of both sexes propagated to 100 m with a stable decrease in SNR (Fig 4). In the hilly forest between the streams, it was not possible to measure song degradation at 100 m from the source. SNR for the STREAM experiment decreased substantially more than for FOREST (9.0 dB) even at the closest, 12.5 m distance. For measurements after 25 m the difference in comparison to the plain forest was vast (14.5 dB). What is interesting is the pattern of degradation was uneven with distance and based on SNR values songs after 50 m were less degraded (by 3 dB) than after 25 m of transmission. We attribute this difference to the micro-location of recording points. The recording at 25 m in STREAM was in a terrain depression while at 50 m it reached roughly the same level as the loudspeaker. This is typical for signal transmitted in heterogenous habitats consisting of a mosaic of patches which may have a contrasting effect on sound signal [9, 42].

In fact, one of the factors that we did not directly test in the current study was the perch height of singing and listening birds. This is the most general method for assessing transmission [43], but obviously it is somewhat of a simplification [9]. The height-dependent degradation within a forest is regarded as an important selection pressure for transmissibility in avian signals, and the terrain variation in mountain forest might only amplify differences which normally occur in level forest [9]. In a mountain forest we have many combinations of signaller and receiver positions which may affect transmission in various ways. The comparison of FOREST and STREAM transmissions suggests that for a mountain forest species inhabiting a wide range of heights above the ground as boubous do, birds have a greater potential for optimisation of signal range by choosing locations. Studies on other forest species like Eurasian Blackbird (*Turdus merula*) [7] and Eurasian Wren (*Troglodytes troglodytes*) [8] have found perch height can be used as a way of aiding propagation. In mountains, such aids through the selection of perch heigh using elevated terrain could be important.

#### Song propagation in deforested habitat

The SHRUBS propagation was conducted in regrowing, burnt forest. Such habitats have completely different vegetation structure than mountain forest, but because of human activity this dominating in many areas that were previously overgrown by the forest. Usually, the untouched forest remnants only remain along streams and the shrubs surrounding such places are still the habitats where Yellow-breasted Boubous occur and breed. According to vegetation structure, the transect in SHRUBS was in a typical area with herbs and bushes covering nearly 100% from the ground to 1.5–3 m height. Above this dense cover only scattered trees might occur. Hence, it is a top open, dense zone with greater micrometeorological instability and winds which usually add a random amplitude fluctuations and scatter sounds, especially during midday [41]. Songs of the Yellow-breasted Boubou propagated in such environments significantly worse than in level terrain forests. After 50 m of propagation, the SNR was the lowest (around ~7.1 dB) in comparison to each forest transect and it was not possible to measure song degradation at 100 m from the source. Also, EA was significantly larger in SHRUBS than in any other forest site (Fig 2). On the other hand, degradation in SHRUBS was predictable between distances as it was for FOREST transmission and opposite to the STREAM experiment.

#### Stream noise effects on song propagation

One more factor to consider with the propagation of signals comes with abiotic factors and associated noise. In African mountain forest, the natural sources of abiotic noise are wind, rain and streams. To our knowledge stronger wind and rain stop birds from singing completely, we studied this aspect with a whole year recording approach on automatic recorders [24]. When regarding stream noise, we have some inherited characteristics of this phenomenon which makes it particularly interesting by the meaning of signal evolution. Firstly, it is widespread in mountains as they are crossed by a grid of streams, hence it is a spatially variable factor. Secondly, streams are also variable in time, some of them appear only during the rainy season while others may persist the whole year, and their noise, reflect rainfall levels with a degree of delay. Although stream noise should have a negative influence on sound transmission, direct surroundings of streams are likely the best habitats for many species because of high productivity and food availability, especially when the forest fragments remain only along streams [44].

The comparison of song degradation between FOREST and STREAM experiments does not allow us to separate the effect of stream noise from terrain effect (Table 2). This also applies to the direct comparison of SHRUBS and STREAM. However, we have conducted two more propagation experiments (STREAM TO and STREAM FROM) which allow for direct comparison of close stream noise, no terrain obstacles and in two combinations of signaller-receiver (here: speaker and microphone) locations. We found that close vicinity to a loud stream has a strong deterioration effect on songs and that it was stronger for wider frequency band sounds like female songs or the male Hwee-hwee song type. We also found that the positions of the signaller and receiver in relation to the source of noise is also very important. Being receiver located close to the source (~2 m) of abiotic noise resulted in ~6 dB higher SNR ratio for songs produced only 25 m apart, in comparison to the converse location. A recent paper by Sueur et al. [45] describes how abiotic factors such as stream noise or wind can affect the transmission of acoustic signals in the environment and that climate change may be causing an increase in these abiotic factors, thereby having an increasing impact on the acoustic signal propagation of various animal species. For example, elephant vocalisations dramatically change throughout a whole day due to abiotic factors of wind and air temperature with certain conditions being optimal and whilst others less so [46]. In the case of the Bamenda Highlands, the main effect of human activity is serious deforestation of the area which simply forces the boubous (and many other species) to inhabit only the narrow forest corridors that are left along streams. Hence, they must live in a habitat with elevated ambient noise.

### Differences in propagation of male and female songs

#### Male songs propagate further

We found that male songs degraded significantly less than female songs in all kinds of tested conditions. Even though we used for the playback songs we transmitted them with the same amplitude for both sexes. The level we used was within the natural range of both males and females, however, these ranges are moderately big, and females usually sing quieter than males, while simultaneously males might sometimes exceed the level used for playback considerably. Therefore, the typical differences in the active range of males and females could be even larger than we are able to indicate with these experiments.

The difference in signal propagation between sexes was something expected based on the knowledge about how different sounds degrade through transmission [2, 4, 47] and how males and females of the Yellow-breasted Boubou sing [22]. The narrow frequency bandwidth characteristic for the High whee-oo and Low whee-oo song types of males, allowed for lower degradation than the wider Hwee-hwee song types, and especially for any female vocalisations. However, the question why males and females sing so differently is not trivial and not easy to answer. Usually, such big differences are shown when conducting between species comparisons, revealing species adaptation for living in acoustically different habitats (e.g. [11]). However, here we only have one species with the two sexes occupying and defending the same territory together [22, 23, 26]. Hence, the causes for the structural differences of songs between the sexes and how this is related to their transmission properties should be biological and linked with functions.

Our study suggests that while singing at the same level of amplitude, the active range of a female song could be half that of a male song in a comparable condition. We also know that males are singing more intensively both as solos and as duet initiators, while females when singing alone usually stop vocalising very quickly [22, 24]. Moreover, male song carries identity information, while we have no support for identity coding in female songs [22, 48] (Szymański et al. under review). All these data suggest that male vocal signals are aimed at potential receivers in at a distance, regardless of whether they are neighbouring pairs, intruders or their own mate. On the other hand, the female song characterised by lower intensity and a quick cease of production when the partner is not responding, fits well to be the signal adjusted especially for within-pair communication. Its faster degradation caused by atonal character and wide frequency range has from this perspective also some advantages from this perspective as it could be used as a cue for ranging as was already found in other tropical forest species [10, 16]. A female responding to a mate may signal the distance to him which, in a visually occluded environment could be important. Synchronized duetting of paired birds that are in close proximity to one another could also be a signal for other birds, allowing for the assessment of joint effort in territory defence, especially as a strong response to strangers was usually found to be a highly synchronised response of pairs [23, 26]. A similar difference has been found for the Rufous-and-white Wren (*Thryophilus rufalbus*) whereby female songs degrade more than male songs [43]. This difference in transmission is thought to relate to the behaviour of the birds, with male vocalisations being used for long range transmission compared to those of the females that are most likely used for close contact communication between the pair [13].

#### Differences in propagation of male song types

We also found some differences in degradation pattern between male song types. In the more demanding habitats like hilly mountain, shrubs or closer to streams, the Hwee-hwee song type degraded more than types High whee-oo and Low whee-oo. This could be explained by the structure of Hwee-hwee songs which are characterised by the larger frequency band and shorter duration of components. Earlier findings indicate some functional differences between male song types. Firstly, the three song types are used among males with consistent regularity (High whee-oo > Low whee-oo > Hwee-hwee) both when produced as solo and in duets [22]. The most rarely produced song Hwee-hwee is simultaneously much more frequently than expected by chance produced as solo during the dawn chorus [22] and most often evokes the strongest response during simulated intrusion [23]. The strongly responding males seem to intentionally match the type of playback when the Hwee-hwee song is used in experiments. On the other hand, the High whee-oo song type seems to play a keep away long-range signal addressed to rivals, while the Low whee-oo song type might be aimed more at a female receiver [23]. Hence, it seems that signals (song types High whee-oo and Low whee-oo) aimed at a distant or harmless receiver have a structure that degrades which is degrading slower than signal (song type Hwee-hwee) that is used in a more aggressive context.

### General conclusions

We found clear differences in the Yellow-breasted Boubou song propagation among different site types reflecting both differentiated vegetation and relief, as well as between the different types of vocalisations they produce. Our results indicate that males’ songs propagated much better than female songs in all habitats. On the other hand, even natural and undisturbed mountain forest habitat seem to be very demanding for sound propagation as terrain and stream noise may have locally strong negative effect on the signal active space. Observation and listening to spontaneously singing Yellow-breasted Boubous allowed us to assess their audibility for at least 300–500 m from a human listener (as measured by GPS while walking away from a bird singing from the same known place). On the other hand, a descent behind the ridge of a hill made it almost impossible to hear a bird singing from under 50 m (own unpublished observations). It seems, therefore, that under such conditions acoustic signals had to be subjected to a particularly strong natural selection. It may work in two ways. Under certain conditions the birds may suffer from a limited propagation range of signals, but on the other hand, they may also use local environment characteristics to enhance signal transmission.

An important question is how deforestation in general may affect the communication system of the species. Within the study area, most available habitats are forest remnants along streams with relatively narrow belts of forest surrounded by shrubs or herbal vegetation. Yellow-breasted Boubous inhabiting such areas stay in the most acoustically demanding environment. Streams produce elevated noise and often flow through ravines, which also limit sound propagation due to hilly terrain. Moreover, the surrounding shrubs cause sound to degrade strongly and limit the possibility of singing from elevated places. Living in such conditions may specifically affect the communication network. Notably, this is also because the areas between streams are covered by shrubs, which has been shown to limit song propagation seriously and flatten the differences between male and female songs’ degradation found in the forest habitats. Results presented in this study indicate that linear distribution of territories along streams may only limit acoustic contact to the direct neighbours. This may contribute to further population fragmentation of the study species. Birds are known to adapt their vocal signals to changes in abiotic conditions which, in turn, can lead to a shift in the acoustic niches of certain species [45]. A change in the acoustic niche can cause bird species to alter the frequency of sound signals, as seen with Great tits (*Parus major)* in urban sites compared to rural areas [49]. However, it is hard to imagine a strategy the Yellow-breasted Boubous may apply for adaptation to the habitat fragmentation in this case.

## Supporting information

S1 Checklist (DOCX)

S1 Fig A photograph illustrating a natural forest located on a relatively level terrain (placement of FOREST transmission experiment). (JPG)

S2 Fig A photograph illustrating typical shrub habitat formed after deforestation (burning) of the primary montane forest habitat (placement of SHRUBS transmission experiment). (JPG)

S3 Fig A photograph illustrating the primary montane forest on a hill between two streams which generated substantial noise (placement of STREAM, STREAM FROM and STREAM TO propagation experiments). (JPG)

S1 Table Results of the general linear mixed models for signal-to-noise ratio (SNR), excess attenuation (EA), and tail-to-signal ration (TSR) of propagated male song types (High whee-oo, Low whee-oo and Hwe-hwee) of the Yellow-breasted Boubou (*Laniarius atroflavus*). Main and two-factor interaction effects are presented. Repeated measurements of songs of the same individuals are included as random factor.
(DOCX)

S1 Data (ZIP)
