# Supplementary figures and images for: Male and female songs propagation in a duetting tropical bird species in its preferred and secondary habitat
Source: PLoS One. 2022 Oct 3;17(10):e0275434. doi: 10.1371/journal.pone.0275434 (PMC9529145; doi:10.1371/journal.pone.0275434)

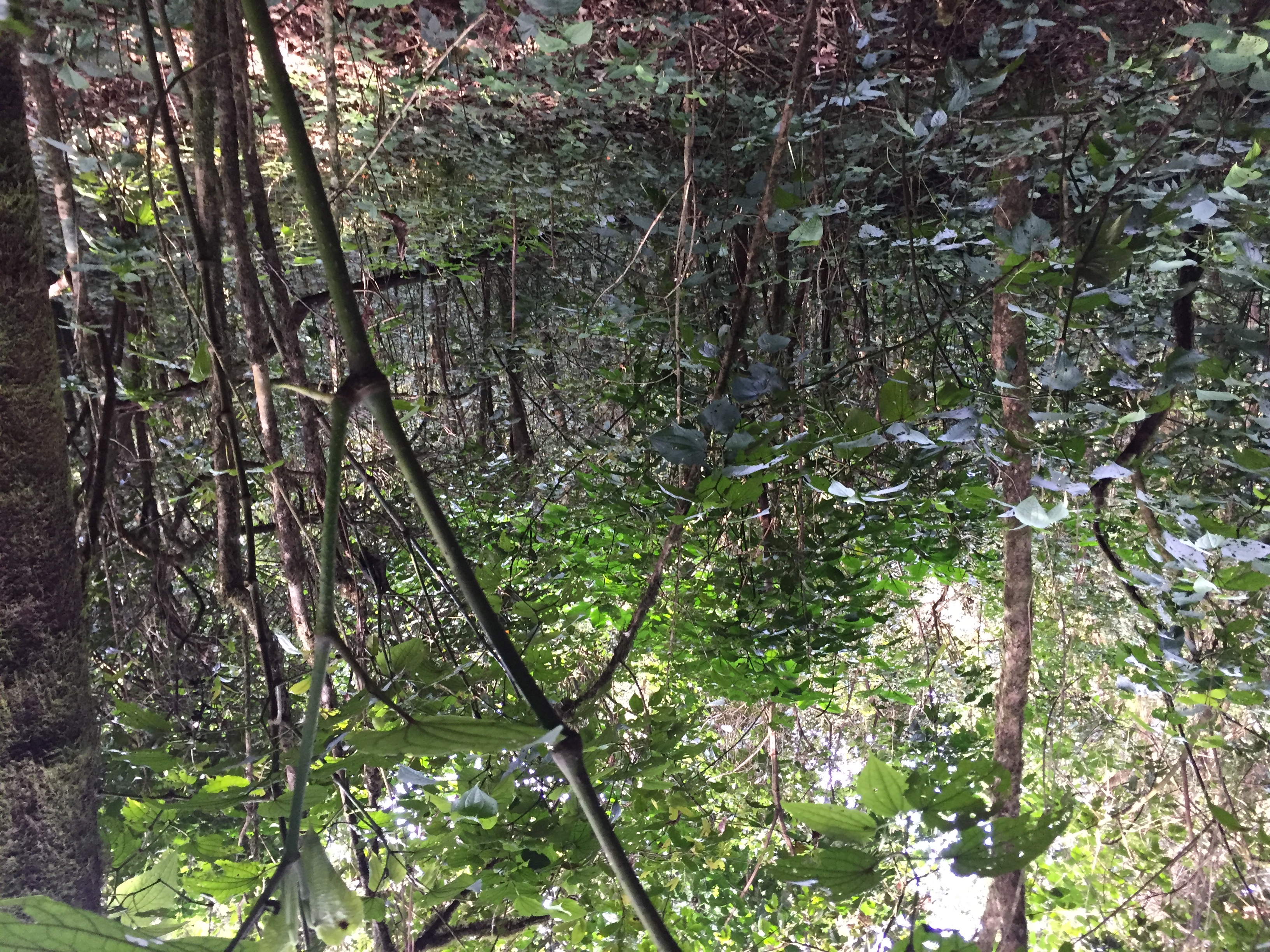

Supplement: S1 Fig — (JPG) [file pone.0275434.s002.JPG]

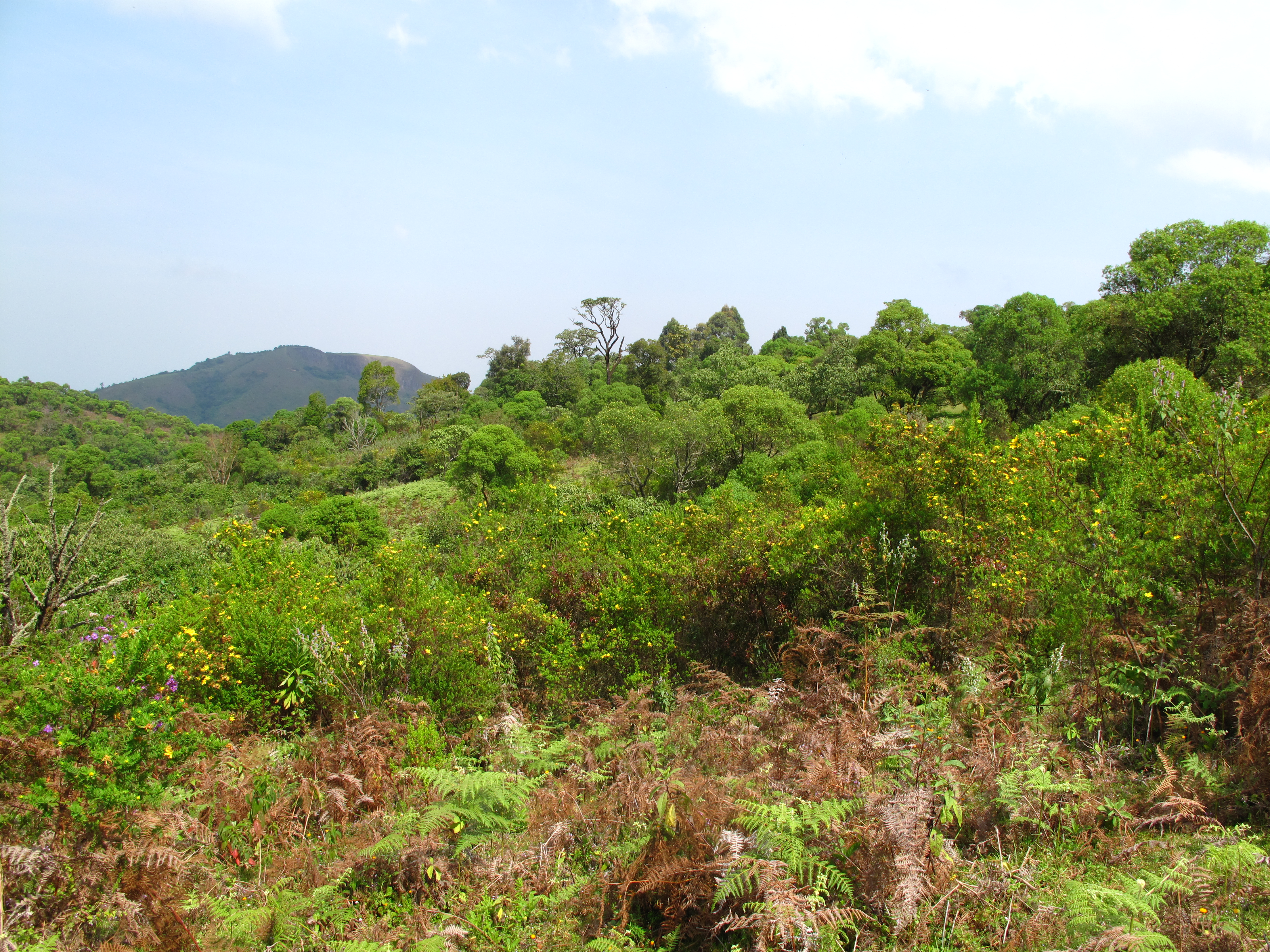

Supplement: S2 Fig — (JPG) [file pone.0275434.s003.JPG]

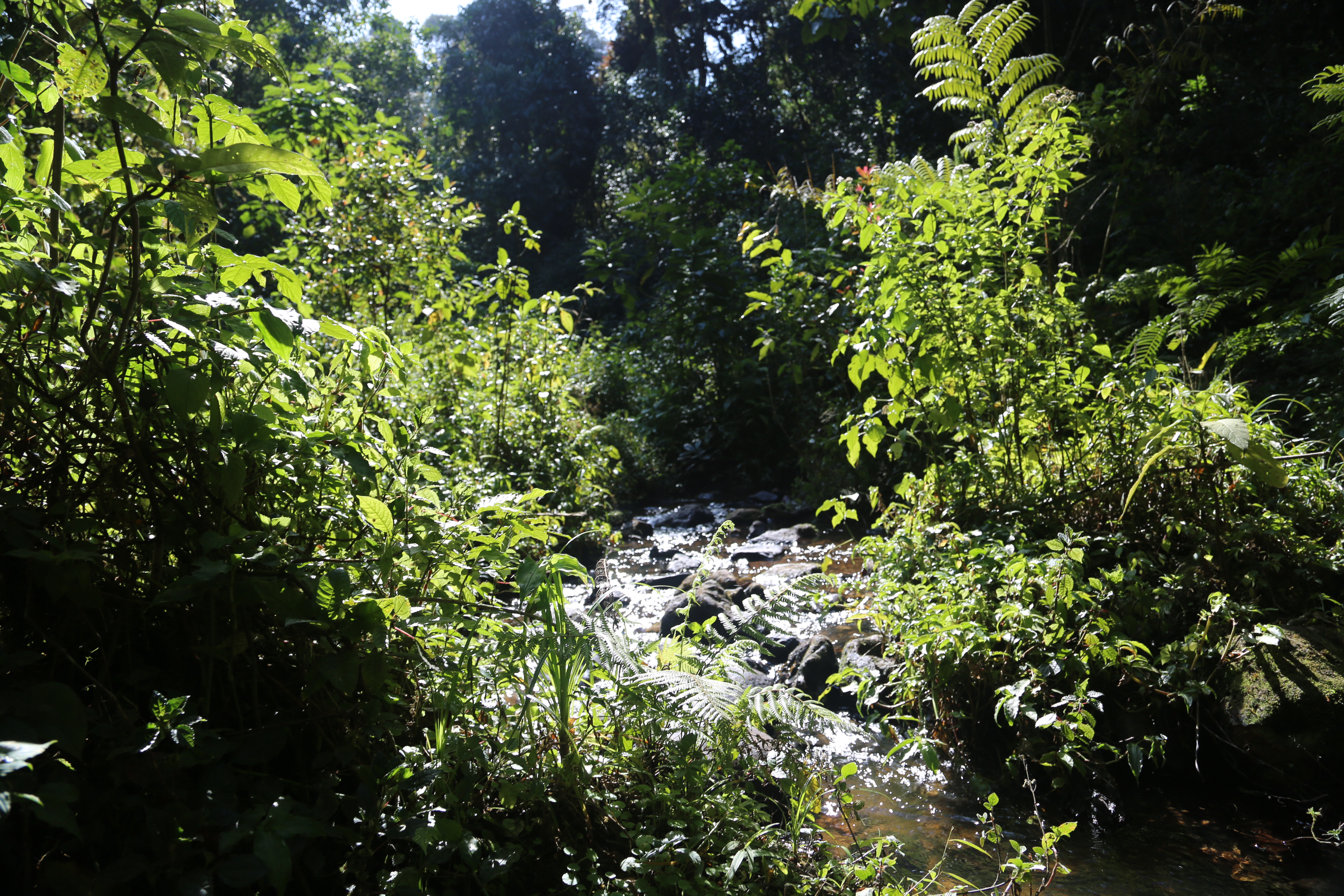

Supplement: S3 Fig — (JPG) [file pone.0275434.s004.JPG]
