# Supplementary material for: Male and female songs propagation in a duetting tropical bird species in its preferred and secondary habitat
Source: PLoS One. 2022 Oct 3;17(10):e0275434. doi: 10.1371/journal.pone.0275434 (PMC9529145; doi:10.1371/journal.pone.0275434)
Supplement: S1 Table — Main and two-factor interaction effects are presented. Repeated measurements of songs of the same individuals are included as random factor. (DOCX) [file pone.0275434.s005.docx]

**Table S1. Results of the general linear mixed models for signal-to-noise ratio (SNR), excess attenuation (EA), and tail-to-signal ration (TSR) of propagated male song types (High whee-oo, Low whee-oo and Hwe-hwee) of the Yellow-breasted Boubou (*Laniarius atroflavus*). Main and two-factor interaction effects are presented. Repeated measurements of songs of the same individuals are included as random factor**.

| Experiment and source of variation | Coef. | Std. Err. | t | P |
| --- | --- | --- | --- | --- |
| **FOREST experiment** | | | | |
| SNR *(dB)* | | | | |
| Male song type | -1.67 | 0.542 | -3.09 | 0.0041 |
| Distance | -7.54 | 0.182 | -41.34 | <0.0001 |
| Male song type × Distance | 0.44 | 0.097 | 4.55 | <0.0001 |
| EA *(dB)* | | | | |
| Male song type | -0.70 | 1.063 | -0.66 | 0.514 |
| Distance | 7.63 | 0.387 | 19.71 | <0.0001 |
| Male song type × Distance | -0.96 | 0.206 | -4.66 | <0.0001 |
| TSR *(dB)* | | | | |
| Male song type | 0.61 | 0.325 | 1.88 | 0.0675 |
| Distance | 2.37 | 0.123 | 19.28 | <0.0001 |
| Male song type × Distance | -0.21 | 0.065 | -3.19 | 0.0014 |
| **SHRUBS experiment** | | | | |
| SNR *(dB)* | | | | |
| Male song type | -2.28 | 0.503 | -4.54 | <0.0001 |
| Distance | -10.16 | 0.346 | -29.35 | <0.0001 |
| Male song type × Distance | 0.15 | 0.182 | 0.84 | 0.401 |
| EA *(dB)* | | | | |
| Male song type | 2.50 | 0.855 | 2.92 | 0.0058 |
| Distance | 10.54 | 0.441 | 23.87 | <0.0001 |
| Male song type × Distance | -1.13 | 0.232 | -4.86 | <0.0001 |
| TSR *(dB)* | | | | |
| Male song type | 2.76 | 0.448 | 6.17 | <0.0001 |
| Distance | 4.97 | 0.279 | 17.80 | <0.0001 |
| Male song type × Distance | -0.87 | 0.146 | -5.98 | <0.0001 |
| **STREAM experiment** | | | | |
| SNR *(dB)* | | | | |
| Male song type | -2.08 | 0.499 | -4.16 | <0.0001 |
| Distance | -4.07 | 0.345 | -11.78 | <0.0001 |
| Male song type × Distance | -0.02 | 0.185 | -0.13 | 0.898 |
| EA *(dB)* | | | | |
| Male song type | -0.71 | 1.051 | -0.67 | 0.507 |
| Distance | -0.08 | 0.302 | -0.27 | 0.788 |
| Male song type × Distance | -0.01 | 0.161 | -0.09 | 0.925 |
| TSR *(dB)* | | | | |
| Male song type | 0.88 | 0.397 | 2.22 | 0.0324 |
| Distance | 1.61 | 0.181 | 8.92 | <0.0001 |
| Male song type × Distance | -0.10 | 0.097 | -1.06 | 0.2898 |
